# Analysis of the SOS response of *Vibrio *and other bacteria with multiple chromosomes

**DOI:** 10.1186/1471-2164-13-58

**Published:** 2012-02-03

**Authors:** Neus Sanchez-Alberola, Susana Campoy, Jordi Barbé, Ivan Erill

**Affiliations:** 1 Departament de Genètica i de Microbiologia, Universitat Autònoma de Barcelona, 08193 Bellaterra, Spain; 2 Department of Biological Sciences, University of Maryland Baltimore County, Baltimore 21228, USA

## Abstract

**Background:**

The SOS response is a well-known regulatory network present in most bacteria and aimed at addressing DNA damage. It has also been linked extensively to stress-induced mutagenesis, virulence and the emergence and dissemination of antibiotic resistance determinants. Recently, the SOS response has been shown to regulate the activity of integrases in the chromosomal superintegrons of the *Vibrionaceae*, which encompasses a wide range of pathogenic species harboring multiple chromosomes. Here we combine *in silico *and *in vitro *techniques to perform a comparative genomics analysis of the SOS regulon in the *Vibrionaceae*, and we extend the methodology to map this transcriptional network in other bacterial species harboring multiple chromosomes.

**Results:**

Our analysis provides the first comprehensive description of the SOS response in a family (*Vibrionaceae*) that includes major human pathogens. It also identifies several previously unreported members of the SOS transcriptional network, including two proteins of unknown function. The analysis of the SOS response in other bacterial species with multiple chromosomes uncovers additional regulon members and reveals that there is a conserved core of SOS genes, and that specialized additions to this basic network take place in different phylogenetic groups. Our results also indicate that across all groups the main elements of the SOS response are always found in the large chromosome, whereas specialized additions are found in the smaller chromosomes and plasmids.

**Conclusions:**

Our findings confirm that the SOS response of the *Vibrionaceae *is strongly linked with pathogenicity and dissemination of antibiotic resistance, and suggest that the characterization of the newly identified members of this regulon could provide key insights into the pathogenesis of *Vibrio*. The persistent location of key SOS genes in the large chromosome across several bacterial groups confirms that the SOS response plays an essential role in these organisms and sheds light into the mechanisms of evolution of global transcriptional networks involved in adaptability and rapid response to environmental changes, suggesting that small chromosomes may act as evolutionary test beds for the rewiring of transcriptional networks.

## Background

Bacteria are frequently exposed to a broad range of stressors that may cause, directly or indirectly, DNA damage. First described in *Escherichia coli*, the SOS response comprises the rapid and concerted activation of a specific set of genes aimed at addressing DNA damage [1,2]. The synchronized activation of SOS genes is mediated by the transcriptional repressor LexA [3]. The *E. coli *LexA protein forms dimers that are able to recognize a palindromic binding motif, commonly referred to as the LexA or SOS box, which has been shown to follow a CTGT-N_8_-ACAG consensus [3,4]. Binding sites conforming tightly to the LexA-binding motif are typically located near or overlapping the RNA-polymerase binding-site on the promoter region of SOS genes [5]. When bound to their target sites, LexA dimers physically hinder RNA-polymerase binding and thereby effectively repress gene expression. The SOS response is induced through the activation of the RecA protein [6,7]. Single-stranded DNA fragments, generated by replication fork stall or enzymatic processing of broken DNA ends [8], bind and activate RecA [9]. In its active form, RecA will induce the auto-catalytic cleavage of the LexA dimer and several other transcriptional repressors, such as the λ phage CI repressor. Cleaved LexA is unable to bind its target sites, thus activating the expression of SOS genes [9]. The *E. coli *SOS transcriptional network comprises more than 40 genes involved in diverse forms of DNA repair, error-prone DNA replication and cell division arrest, and it includes also both the *recA *and *lexA *genes [5,10,11]. Once DNA damage has been addressed, activated RecA levels fall rapidly and newly synthesized LexA returns the system to its repressed state.

The SOS response was first identified in response to UV treatment, but it is also intrinsically linked to the natural response against antibiotics that damage DNA [12]. Furthermore, the need to bypass lesions that cannot be readily repaired using translesion synthesis polymerases (TLS) establishes the SOS response as a de facto mechanism for stress-induced mutagenesis [13-15]. In addition, the SOS response has long been known to promote prophage induction [16] and the activation of virulence factors, like Shiga-like toxins in enterohemorrhagic *E. coli *[17]. Independent studies have shown that activation of the SOS response also triggers the dissemination of other mobile elements, such as pathogenicity islands [18]. Finally, it has been reported that several antibiotics that do not cause direct damage to bacterial DNA (e.g. β-lactams) are capable of inducing the SOS response through indirect routes [19], and that such induction is capable of triggering dissemination of mobile elements carrying antibiotic resistance determinants [20]. These findings have irrevocably transformed the image of the SOS response from a DNA-repair textbook paradigm into a key component in the fight against virulence and antibiotic resistance [21-23].

The recent evidence for a tight link between dissemination of antibiotic resistance, pathogenicity and the SOS response is perhaps most clearly outlined in the genus *Vibrio*. This group of heterotrophic and aerobic γ-Proteobacteria encompasses at least 12 species known for being pathogenic in human hosts [24]. *Vibrio *species are usually linked to intestinal infections (e.g. *Vibrio cholerae *or *Vibrio parahaemolyticus*) acquired by drinking of contaminated water or eating of raw sea animals [25-27], but they are also well documented as opportunistic pathogens associated with high morbidity rates (e.g. *Vibrio alginolyticus *or *Vibrio vulnificus*) [28-30]. The genome of *Vibrio *species is composed of 2 circular chromosomes (known as large/1 and small/2) and in most cases it is known to harbor several virulence determinants, including toxins and colonization factors, that are strongly linked to the well-known severity and harshness of classical *Vibrio *infections, such as cholera [31-33]. In 2004, Beaber *et al. *showed that transfer of the *V. cholerae *SXT integrative-conjugative element (ICE) was controlled by the SOS response through RecA-dependent cleavage of the SXT SetR repressor [34]. The SXT is a 100 kbp element encoding genes that confer resistance to several antibiotics, such as chloramphenicol, sulphamethoxazole or trimethoprim, and is now found in almost all clinical isolates of *V. cholerae *from Asia and Africa [35]. The fact that SOS-mediated activation of SXT conjugation could be induced by antibiotics, such as ciprofloxacin, put forward for the first time a mechanism by which the use of antibiotic agents might promote the dissemination of antibiotic resistance determinants [34]. Soon thereafter, Quinones *et al. *showed that induction of the CTX prophage, which encodes the cholera toxin, is regulated by the SOS repressor LexA in conjunction with the phage repressor RstR, and the same group recently proved that transcription of the *rstR *gene is also regulated by LexA [36,37]. Most recently, the SOS response has been shown to regulate the activity of the integrase gene of the *V. cholerae *superintegron, leading to heightened rates of recombination excision of integron cassettes upon induction of the SOS response by different antibiotics [38]. The *V. cholerae *superintegron encompasses more than 150 gene cassettes and is known to be a source of genetic diversity and antibiotic resistance genes [39,40]. SOS regulation of the integrase gene has later been confirmed in chromosomal superintegrons of the *Vibrio *genus and in most mobile integron classes, establishing a mechanism for the long-term safe storage of otherwise harmful adaptive traits, such as antibiotic resistance determinants [41].

Since its initial description in *E. coli*, the SOS response has been described in many other bacteria and, with some notable exceptions (e.g. the ε-Proteobacteria), it is considered a universal stress response in the Bacteria domain [23]. In contrast to other transcriptional networks, like the *arg *regulon [42], the SOS response has undergone extensive evolutionary changes in the binding motif recognized by the repressor protein LexA. As a consequence, the original description of a CTGT-N_8_-ACAG binding motif in *E. coli *has been extended to most β- and γ-Proteobacteria species [43,44], but has been replaced by alternative descriptions in many different classes and phyla. The universality of the *E. coli *LexA-binding motif was first challenged by the description of a GAAC-N_4_-GTTC LexA-binding motif in the Firmicutes *Bacillus subtilis *[45]. This new motif was later confirmed to be the LexA-binding motif in the Actinobacterium *Mycobacterium tuberculosis *and, by extension, in all Gram-positive bacteria [46]. Following this initial discovery, research has unearthed a plethora of LexA-binding motifs in different bacterial groups, ranging from a GTTC-N_7_-GTTC direct repeat in the α-Proteobacteria to a TTAC-N_3_-GTAA palindrome in *Bdellovibrio bacteriovorus *[47,48]. The identification of the LexA-binding motif in several bacterial groups has led to the outlining of a loosely conserved core of genes that seem to constitute the core or ancestral LexA regulon. These include the two main SOS genes (*lexA *and *recA*), members of the nucleotide excision repair (NER) system (most notably the excinuclease subunit A, *uvrA*) and several TLS polymerases [23]. Recently, the canonical view of the SOS response as a DNA repair system has shifted considerably towards a mutagenesis-centered perspective following the identification of a mutagenesis cassette (*imuA*-*imuB*-*dnaE2*) that is widely distributed in the Bacteria domain. Whenever present, this cassette is persistently regulated by LexA, being the only transcriptional unit regulated by the SOS repressor in several organisms [15,48-50].

The rapid increase in the number of completely sequenced bacterial genome sequences in the early 2000 s led to the development of comparative genomics approaches for the analysis of transcriptional networks [42,51-53]. The main premise in these approaches is that binding sites for a transcription factor, if functional, will tend to be preserved by evolution. Hence, the analysis of multiple genomes will identify functional binding sites as conserved regions across several genomes, in spite of significant changes to their immediate neighborhood. Functional conservation of binding sites can be exploited in two different ways. In motif discovery applications, the upstream regions of orthologous genes can be scanned to identify consistent instances of a candidate binding motif [54-56]. In contrast, transcriptional regulatory network analyses use a known binding motif to search multiple genomes and elucidate the composition of the regulatory network at different levels [42,57,58]. In this approach, the identification of a candidate binding site upstream of an ortholog in different species bolsters the a priori low confidence of the *in silico *prediction made on each individual genome [59,60].

A basic tenet of comparative genomic approaches for regulon analysis is the assumption that the transcription factor-binding motif is conserved across the species being studied. In spite of the apparent diversity in LexA-binding motifs across the Bacteria domain, research on different phyla has also shown that changes in LexA-binding motif tend to be monophyletic for the clades in which they take place [61-63]. This has enabled research into the composition and evolution of the SOS response in several bacterial groups using comparative genomics approaches. These studies have revealed that, beyond the conserved core outlined above, the genetic composition of the SOS response can vary substantially between and within Proteobacteria classes [23,43,64]. Because the original collection of binding sites may not belong to any of the species under analysis, a good practice in comparative genomics involves the experimental validation of *in silico *predictions in at least one of the organisms under study, in order to validate the computational method and the parameters used for the analysis [64].

In this work, we take advantage of previously developed methodology to map for the first time the SOS regulatory network in the *Vibrionaceae *family. We apply computational analyses to the 14 complete genome sequences available for this family, which encompass nine different *Vibrionaceae *species, and we validate our findings *in vitro *using *V. parahaemolyticus *as an experimental model. Our analysis thus provides a first glimpse at the genetic organization of the SOS response in a complex and heterogeneous family of bacteria comprising more than a hundred species. In order to gain insight into the adaptation of the SOS response to genomes containing multiple chromosomes, which are a defining feature of the *Vibrionaceae*, we extend the comparative genomics analysis to two additional phylogenetic groups (the β- and α-Proteobacteria) that contain many species with multiple chromosomes. Our results provide comprehensive support to the notion that the SOS response is strongly associated with dissemination of antibiotic resistance and pathogenicity in the *Vibrio *genus, and identify new genes that might play a role in this association. We also show that the core constituents of the SOS response in all the groups analyzed are located systematically in the large chromosome, indicating that the SOS response is a fundamental component of these genomes. The observed distribution of non-core genes among the smaller chromosomes and plasmids puts forward a mechanism for the dynamic evolution of a critical response network involved in the regulation of many essential genes, and supports the hypothesis of plasmid origin for the smaller chromosomes of organisms with multiple chromosome genomes.

## Results and Discussion

### The SOS regulon of the *Vibrionaceae *encompasses more than 20 genes

**Figure 1:**
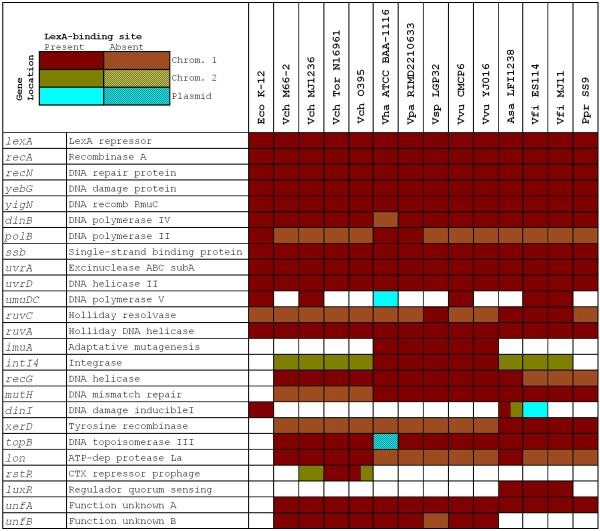
**Tabulated description of the LexA regulon of the *Vibrionaceae***. Colors indicate the presence and location of the gene and patterns denote presence (plain) or absence (patterned) of one or more LexA-binding sites in its promoter region. *E. coli *genes and their corresponding regulation are shown for comparative purposes. Abbreviations are as follows: Eco, *E. coli*; Vch, *V. cholerae*; Vha, *V. harveyi*; Vpa, *V. parahaemolyticus*; Vsp, *Vibrio splendidus*; Vvu, *V. vulnificus*; Asa, *A. salmonicida*; Vfi, *V. fischeri*; Ppr, *Photobacterium profundum*.

**Figure 2:**
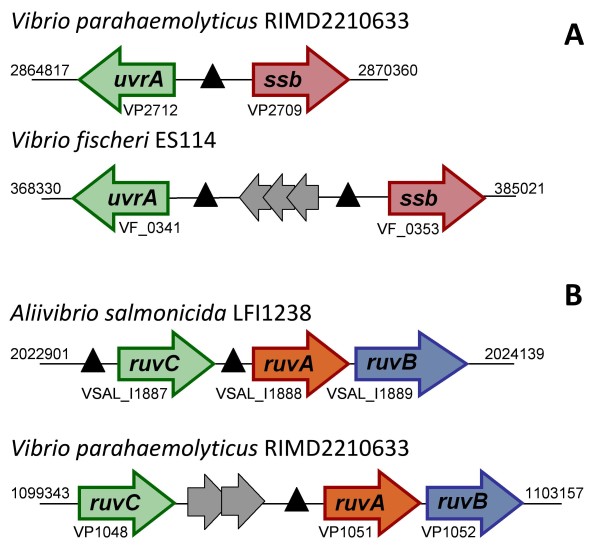
**Schematic representation of the *uvrA*-*ssb *divergent pair and the *ruvCAB *operon in different *Vibrionaceae *species**. The figure illustrates the persistence of *ssb *regulation despite gene insertions and the disruption of the *ruvCAB *operon by gene insertions in some *Vibrio *species. Genes are represented by filled arrows. Grey-filled arrows correspond to genes that do not constitute part of the canonical *uvrA*-*ssb *and *ruvCAB *elements. Black triangles indicate predicted LexA-binding sites. GenBank accession numbers and absolute genome coordinates for each bacterial species are provided for reference.

To analyze the SOS regulon of the *Vibrionaceae*, we downloaded all the available complete genome sequences from the NCBI RefSeq database [65] and we performed independent searches of both chromosomes and sequenced plasmids using a collection of known binding sites from *E. coli *[43]. As in previous work, to define the family-wide SOS regulon we included only putative sites that were observed consistently upstream of orthologous genes in at least three separate species. The results of the comparative genomics analysis shown in Figure 1 suggest that, in accordance with other LexA regulons, the SOS response of the *Vibrionaceae *comprises more than 20 genes [5,66]. Given the relative phylogenetic proximity between the *Vibrionaceae *and *E. coli*, it is not surprising to find that many of the genes described as SOS regulated in *E. coli *are also members of the *Vibrionaceae *SOS regulon. These include the two main regulators of the SOS network (*lexA *and *recA*), as well as canonical SOS genes involved in DNA repair (*recN, yebG, yigN, uvrA, uvrD *and the *ruvAB *operon) and translesion synthesis (*dinB *and *umuDC*). Nonetheless, differences in the mode of regulation of certain genes can be observed in the *Vibrionaceae*. The *uvrA *gene forms a divergent gene pair with *ssb *in *E. coli *and other γ-Proteobacteria, sharing a single LexA-binding site [43]. Expression of both genes has been shown to be SOS regulated, even though SOS induction of *ssb *is known to be moderate [67,68]. This arrangement (Figure 2A) is preserved in many *Vibrionaceae *(e.g. *V. parahaemolyticus*), but has been disrupted by a bacteriophage insertion in other species (e.g. *Vibrio fischeri*). The fact that independent LexA-binding sites for *ssb *and *uvrA *have been preserved in these species points to a selective advantage in the regulation of both genes by the SOS response. In contrast, the *ruvCAB *operon yields a different story (Figure 2B). In *E. coli *and close relatives, this operon is split into the *ruvC *gene and the *ruvAB *operon, and only the latter is regulated by the SOS response [5,43]. In *Aliivibrio salmonicida *and other species, the operon retains its ancestral *ruvCAB *configuration but displays independent LexA-binding sites upstream of both *ruvC *and the *ruvAB *tandem, which presumably incorporates a secondary promoter. The operon has later been disrupted by the insertion of one (*V. vulnificus*) or two (*V. parahaemolyticus*) genes and the regulation of *ruvC *has apparently been lost in the process. This suggests that the selective pressure for SOS regulation of *ruvC *is not as strong as the one operating on *ruvAB*, in agreement with recent results that emphasize the independent and vital role of the *ruvAB *complex in rescuing stalled replication forks [69].

The comparative genomics analysis also identifies putative binding sites upstream of several genes that are not members of the *E. coli *LexA regulon but that have been shown experimentally to be SOS regulated in at least one of the species under analysis. The promoter of the *luxR *gene of *V. fischeri*, for instance, has been shown to bind LexA and its own product (LuxR), which share the same binding site [70,71], and our analysis indicates that this cross-regulation is maintained in the closely related *A. salmonicida*. Similarly, we identify LexA-binding sites in the promoter of the aforementioned *rstR *gene in all the *V. cholerae *strains harboring the CTX prophage [37]. Close examination of these promoter regions in classical and El Tor biovars reveals that the LexA binding site mediating the regulation of *rstR *has been selectively maintained in spite of substantial changes to its promoter region [72]. In CTX phages of the classical biotype, the region encompassing the LexA-binding site contains several insertions with respect to El Tor biotypes, in which the combined RstR-LexA regulation has been analyzed in detail [37] (Additional file 1). These insertions, together with the specific sequence of the LexA-binding site, can in fact be used reliably to determine the CTX biotype of *Vibrio *sequences. The variability of the *rstR *promoter is in contrast with that observed in other SOS regulated elements of *V. cholerae*. For instance, the promoter of the *setR *gene in the integrating-conjugative element SXT shows remarkable conservation of promoter elements and SetR-binding sites among strains (Additional file 2). Significant conservation is also observed within each biotype for the *rstR *promoter, suggesting that both variants of CTX and the SXT element have spread recently among *V. cholerae *strains. Our analysis also uncovers the recently reported regulation of *Vibrio *superintegron integrase genes [38,41] and the systematic regulation of the *imuA*-*imuB*-*dnaE2 *mutagenesis cassette in all the species in which it is present [49]. Interestingly, the presence of this mutagenesis cassette correlates neatly with the location of the superintegron (found in the large chromosome in all the species harboring the cassette and in the small chromosome otherwise). This suggests that both traits are the product of a single reorganization event that took place before the split of *V. cholerae *from other *Vibrionaceae*, (such as *Vibrio harveyi*), a fact that is in consonance with the existing 16 S rRNA phylogeny for this family [32].

### LexA binds predicted SOS genes in *V. parahaemolyticus*

**Figure 3:**
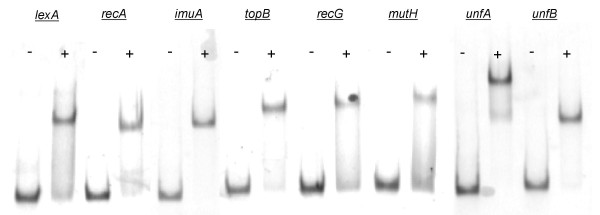
**EMSA of the promoter region for several *V. parahaemolyticus *genes in the presence (+) or absence (-) of purified LexA protein from this organism**. Competition assays (Additional file 3) were conducted to validate the specificity of LexA binding. The absence of purified LexA protein is used as a negative control for each EMSA. The *lexA, recA * and *imuA * promoter regions are used as positive controls.

To validate the results of the *in silico *comparative genomics analysis, we performed electro-mobility shift assays (EMSA) of putatively regulated promoters of *V. parahaemolyticus *using the purified LexA protein of this organism. EMSA were performed on promoters of three canonical SOS genes (*lexA, recA *and *imuA*) for positive control, and on the promoters of genes flagged as candidate new members of the LexA regulon in the comparative genomics analysis: *topB, recG, mutH *and the two genes coding for proteins of unknown function (*unfA *[VP1264] and *unfB *[VP1428]). The results of the EMSA shown in Figure 3 confirm that the putative LexA-binding sites identified in the comparative genomics analysis are bound by LexA in *V. parahaemolyticus*, thus providing experimental validation for the *in silico *approach. Furthermore, competition assays establish that LexA-binding to these sites is specific (Additional file 3). Hence, even though they do not constitute conclusive proof of SOS regulation, these results provide direct evidence of LexA-binding to the promoter region of five genes that have never been described previously as members of the SOS response. The apparent LexA regulation of *recG *makes intuitive sense. Even though it is not LexA regulated in *E. coli*, the product of *recG *is involved in the generation of Holliday junctions after replication fork stall and is known to interact with other members of the DNA recombination repair system [73]. The same logic applies to *topB*, which encodes a DNA topoisomerase III that has been shown *in vitro *to interact in *E. coli *with the products of *ssb *and *recQ *in the resolution of converging replication forks [74].

The putative SOS regulation of *mutH*, which encodes the DNA mismatch repair (MMR) endonuclease MutH, is more unexpected than that of *recG *or *topB *because no members of the MMR system have been reported to be SOS regulated previously. However, increased concentrations of MutH have been shown to lead to a predominance of MMR over very short patch repair (VSP), mediated by the Vsr endonuclease [75], and prevalence of MMR over VSP may be desirable to minimize the mutagenic effects of VSP. Finally, the *V. parahaemolyticus *EMSA confirm binding of LexA to the promoters of two genes coding for proteins of unknown function (VP1264 and VP1428). Homologs of the VP1264 gene, here termed *unfA*, have been annotated as members of the superfamily II of DNA and RNA helicases, for which *recQ *is the most well-known representative in bacteria. Hence, binding of LexA to the *unfA *promoter in the *Vibrionaceae *might be linked to the regulation of *topB *reported above and is likely to be involved in DNA repair processes. The gene VP1428 (*unfB*), however, has no known homologs outside the *Vibrionaceae *and its product is consistently annotated as hypothetical in all the *Vibrio *species analyzed here. Importantly, *unfB *appears to be present and associated with a putative LexA-binding site only in those *Vibrio *species that are known to be human pathogens. Given the well-founded relationship of the SOS response with dissemination of antibiotic resistance and pathogenicity in the *Vibrio *genus, it seems reasonable to postulate that the *unfB *gene product may be involved in such processes in *Vibrio *species.

### The SOS regulon of Proteobacteria shares a small set of genes

Multiple chromosome genomes have been described and appear to have evolved independently in at least five different bacterial clades [31,76-79]. Besides the γ-Proteobacteria, to which the *Vibrionaceae *belong, complete genome sequences composed of multiple chromosomes are available for the α-Proteobacteria (*Rhizobiaceae, Brucellaceae *and *Rhodobacteraceae *families), the β-Proteobacteria (*Burkholderiaceae *and *Comamonadaceae *families), the Chloroflexi (*Sphaerobacteraceae*), the Deinococci (*Deinococcaceae*) and the Spirochaetes (*Leptospiraceae*). Having validated the comparative genomics approach in the *Vibrionaceae*, we decided to extend the analysis to other phylogenetic groups that present genomes with multiple chromosomes in order to analyze the adaptation of a complex genetic network, like the SOS response, to such genomic environments. The lack of a well defined LexA-binding motif and/or more than one complete genome sequence within a given phylogenetic group restricted our analysis to the β-Proteobacteria, to which the here-validated LexA-binding motif can be applied [43] and to the α-Proteobacteria, in which a suitable LexA-binding motif has already been experimentally validated for comparative genomic approaches [64].

**Figure 4:**
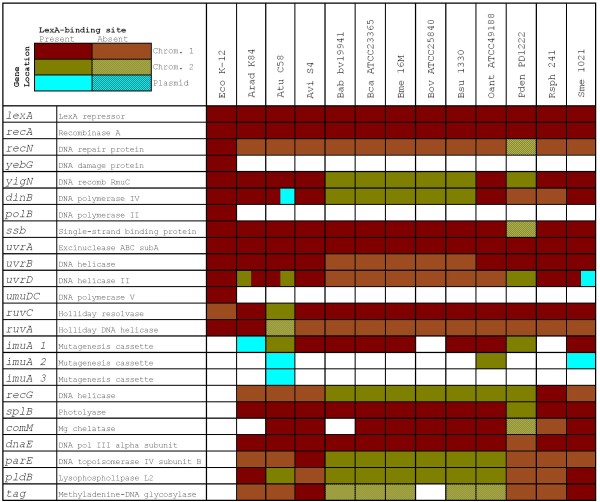
**Tabulated description of the predicted LexA regulon of α-Proteobacteria species with multiple chromosome genomes**. Colors indicate the presence and location of the gene and patterns denote presence (plain) or absence (patterned) of one or more LexA-binding sites in its promoter region. *E. coli *genes and their corresponding regulation are shown for comparative purposes. Eco, *E. coli*; Arad, *Agrobacterium radiobacter*; Atu, *A. tumefaciens*; Avi, *Agrobacterium vitis*; Bab, *Brucella abortus*; Bca, *Brucella canis*; Bme, *Brucella melitensis*; Bov, *Brucella ovis*; Bsu, *Brucella suis*; Oant, *Ochrobactrum anthropi*; Pden, *Paracoccus denitrificans*; Rsph, *Rhodobacter sphaeroides*; Sme, *S. meliloti*.

**Figure 5:**
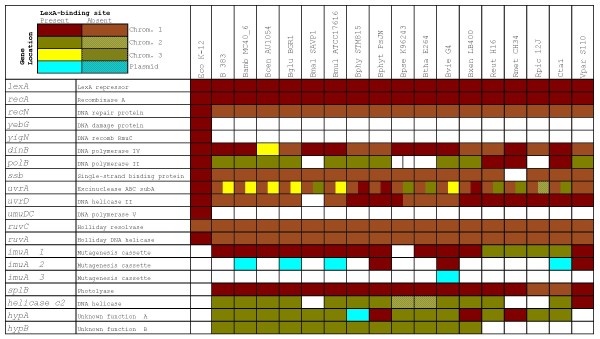
**Tabulated description of the predicted LexA regulon of β-Proteobacteria species with multiple chromosome genomes**. Colors indicate the presence and location of the gene and patterns denote presence (plain) or absence (patterned) of one or more LexA-binding sites in its promoter region. *E. coli *genes and their corresponding regulation are shown for comparative purposes. Eco, *E. coli*; B. 383, *Burkholderia sp*.; Bamb, *Burkholderia ambifaria*; Bcen, *Burkholderia cenocepacia*; Bglu, *Burkholderia glumae*; Bmal, *Burkholderia mallei*; Bmul, *Burkholderia multivorans*; Bphy, *Burkholderia phymatum*; Bphyt, *Burkholderia phytofirmans*; Bpse, *Burkholderia pseudomallei*; Btha, *Burkholderia thailandensis*; Bvie, *Burkholderia vietnamiensis*; Bxen, *Burkholderia xenovorans*; Reut, *Ralstonia eutropha*; Rmet, *Ralstonia metallidurans*; Rpic, *Ralstonia pickettii*; Ctai, *Cupriavidus taiwanensis*; Vpar, *Variovorax paradoxus*.

The results of the comparative genomics analysis for α- and β-Proteobacteria are presented, respectively, in Figure 4 and Figure 5. These support previous results reporting significant variation in the composition of the SOS system across bacterial groups [64,66]. In particular, the results on α- and β-Proteobacteria reveal a conserved core for the SOS regulon that comprises only the *lexA *and *recA *genes, an inducible TLS polymerase (*dinB *and/or *polB*), the NER excinuclease subunit A (*uvrA*) and the mutagenesis cassette *imuA*-*imuB*-*dnaE2*. Beyond this small core, the three phylogenetic groups analyzed here present numerous differences and some relevant similarities. A feature common to α- and β-Proteobacteria is the lack of LexA regulation of the *recN *gene, which is heavily regulated (up to three LexA-binding sites) in *E. coli *and in the *Vibrionaceae*, and which had been formerly identified as a key component of the SOS response [43]. On the other hand, the *recG *gene of some α-Proteobacteria appears to be regulated by LexA, suggesting that the presence of LexA-binding sites upstream of *recG *reported in the *Vibrionaceae *might be due to an ancestral regulation of this gene. The same reasoning can be applied in the case of the *ruvCAB *operon, the promoter of which harbors putative LexA-binding sites in the α-Proteobacteria in spite of substantial genomic rearrangements. In a similar vein, the identification of putative LexA-binding sites in the promoter of β-Proteobacteria Helicase c2 coding genes is congruent with the apparent regulation of *unfA *and *recG *in the *Vibrionaceae*.

### Photoreactivation is under SOS regulation in the α- and β-Proteobacteria

In the α-Proteobacteria, the comparative genomics analysis identifies several genes not belonging to the shared SOS core that have already been reported as SOS regulated in some species with single chromosomes (*parE *in *Sinorhizobium meliloti *and *tag, comM *and *dnaE *in *Caulobacter crescentus*) [64,80]. Conversely, the analysis of the β-Proteobacteria suggests that two genes coding for hypothetical proteins might be LexA regulated in this class. One of them (*hypA*) has no homologs outside β-Proteobacteria species with multiple chromosomes, whereas the other (*hypB*) appears to be exclusive to the *Burkholderia *genus. Beyond the consistent lack of *recN *regulation, however, the predicted α- and β-Proteobacteria SOS networks share an additional trait that is not observed in the γ-Proteobacteria: the apparent regulation of a gene coding for a predicted photolyase (*splB*). The *splB *gene identified here codes for a protein homologous to the spore photoproduct lyase of *B. subtilis*, which is known to address DNA lesions generated by UV radiation in *B. subtilis *spores [81]. The fact that the homologs of this gene present LexA-binding sites in two groups where LexA recognizes markedly divergent LexA-binding motifs suggests that the *splB *gene is a functional member of the SOS response in both groups. This finding is remarkable because it is the first evidence of a photoreactivation enzyme regulated by the SOS response. Together with the predicted regulation of *mutH *in *V. parahaemolyticus*, it extends the conventional reach of this transcriptional response to include the direct regulation of MMR and photoreactivation components.

The apparent regulation of *splB *by the SOS response also points to an adaptive component in the specialization of this transcriptional network in different bacterial groups. As opposed to the *Vibrionaceae*, α- and β-Proteobacteria are mainly soil bacteria [82]. Soil habitats can lead to bacteria being exposed to high levels of UV light in combination with dehydration. This condition is encountered often by *B. subtilis *spores, and the ensuing DNA lesions are repaired by SlpB [81]. Hence, it seems plausible that α- and β-Proteobacteria might benefit from regulation of photoreactivation processes by the SOS response. The case of the *splB *gene thus provides a window into the process of adaptation of this transcriptional network to different ecological niches. More generally, the three LexA regulons analyzed in this work show evidence of radial divergence from a small shared core, and this divergent process has endowed each group with a different set of specialized SOS genes. The cooption of the SOS response to regulate dissemination of antibiotic resistance, pathogenicity and virulence determinants and its enhanced repertoire of repair genes in the *Vibrionaceae *has already been discussed. Presumably, the loss of regulation for some repair genes (e.g. *ruvCAB*) in the β-Proteobacteria and the regulation of DNA glycosilases (*tag*) and competence genes (*comM*) follows a similar, if not so obvious, adaptive rationale.

### The SOS regulon core is contained within the larger chromosome

A main result of the comparative genomics analysis over several phylogenetic groups containing species with multiple chromosome genomes is the observation that the core genes of the SOS response are found predominantly in the large chromosome. Genomes with multiple chromosomes may originate through different mechanisms (e.g. chromosome duplication or partition), but the prevailing view on the evolution of multiple chromosomes in bacteria points to a plasmid origin of the smaller chromosomes [83]. This is supported by the presence of plasmid-like origins of replication in several small chromosomes [83], and by the greater proportion of housekeeping genes, synteny and gene conservation in the large chromosomes [84,85]. Few comparative genomics studies have analyzed regulatory networks in species with multiple chromosomes and none of them has done so in a systematic way. Nonetheless, the scant data available suggest that genes belonging to primary regulatory networks, like those involved in anaerobic respiration, tend to reside in the large chromosome [86], whereas the genes composing more specialized regulons (like the KdgR regulon involved in pectin catabolism) are located mainly in the small chromosomes [57].

The comparative genomics analysis conducted here is thus the first systematic study of the distribution of a regulatory network in genomes with multiple chromosomes. The near-universality of the SOS response in the Bacteria domain and the well-documented housekeeping role of many of its members (e.g. *recA*) [23,87] establish it as an essential component of the genetic makeup of most bacteria. Hence, our analysis provides sound support to the hypothesis that the genomic distribution of primary regulons in species with multiple chromosomes is predominantly skewed towards the large chromosome. On the other hand, our study reveals also that family-specific additions to the SOS network, as well as duplications of core SOS genes, are most frequently located in small chromosomes and plasmids. The genetic content of small chromosomes has been shown recently to evolve faster, putting forward the notion that these secondary chromosomes act as evolutionary test beds subject to reduced purifying selection [88]. Here we build up on this idea to suggest that small chromosomes and plasmids might provide ideal grounds for the rewiring of transcriptional networks and, specifically, for systems like the SOS response that must strike a delicate balance between regulation of essential genes and rapid adaptability to ecological changes.

## Conclusions

This work provides for the first time a detailed description of the SOS regulatory network in a bacterial family of clinical importance. This analysis identifies and experimentally validates new genes bound by LexA in the *Vibrionaceae *and supports the previously reported link between this stress response, the dissemination of antibiotic resistance and the adaptation of *Vibrio *species to human pathogenicity. The mapping of the SOS response in other bacterial groups with multiple chromosome genomes reveals a pattern of recent adaptation to specific environmental niches. Furthermore, it shows that the key elements of this response are consistently located in the large chromosome and it suggests smaller chromosomes and plasmids may serve as test beds for the rewiring of transcriptional networks.

## Methods

### Genome and LexA-binding data

Bacterial species with completely sequenced genomes composed of multiple chromosomes were identified through filtered queries on the NCBI Entrez system [89]. Complete chromosome and plasmid sequences for species with multiple chromosome genomes were downloaded from the RefSeq database in GenBank (.gbk) format [65]. Collections of binding sites for the γ/β- and the α-Proteobacteria were derived from experimental data from *E. coli *and several α-Proteobacteria species as described previously [43,64].

### Comparative genomics analyses

Comparative genomics analyses were carried out using xFITOM, a program for binding site search in genomic sequences [59,90]. All chromosome sequences were searched for putative LexA-binding sites using the *R_i _*index [91] and a motif-normalized threshold as reported previously [92]. Putative LexA-sites identified in a single species were considered candidate sites if they were located within -300 to +50 bp of a gene translation start point and their score was larger than the average score of the original collection minus one standard deviation, as described previously [64]. Gene homology was established on the basis of best reciprocal BLAST hits using BLASTP [93]. A gene was considered to possess a reliable LexA-binding site within a given family if candidate sites were located upstream of its homologs in at least three different species. The detailed results for the searches in the three phylogenetic groups analyzed in this work can be found in Additional file 4.

### Electrophoresis mobility shift assays (EMSA)

The *V. parahaemolyticus lexA *gene was amplified using suitable primers (Additional file 5) and cloned into a pET15b vector (Additional file 6). The *V. parahaemolyticus *LexA protein was over-expressed and purified as described previously [41]. DNA probes were constructed using two complementary 61 bp synthetic oligonucleotides (Additional file 6). EMSA experiments were performed as reported previously [49], using 40 nM of *V. parahaemolyticus *LexA and 20 ng of each DIG-marked DNA probe in the binding mixture. For EMSA competitive assays (Additional file 1), 200 fold of either specific or non-specific non-labeled DNA was added to the binding mixture. In all cases samples were loaded in 6% non-denaturing Tris-glycine poly-acrylamide gel. Digoxigenin-labeled DNA-protein complexes were detected following the manufacturer's protocol (*Roche*).

## Authors' contributions

NSA performed the protein purification and mobility-shift assays, carried out searches for LexA-binding sites and integrated the data. JB, SC and IE conceived the study and participated in its design and coordination. SC designed and directed the *in vitro *studies. IE coordinated the study, designed and supervised the *in silico *analyses, supervised the *in vitro *studies, curated the data and drafted the manuscript. All authors read and approved the final manuscript.

## Supplementary Material

Additional file 1 **Analysis of *rstR *promoter regions**. (A) Comparison of the *rstR*-*rstA *intergenic region of *V. cholerae *O395 classical O1 serotype and *V. cholerae *O1 biovar El Tor str. N16961. Predicted (O395) and known (N16961) promoter elements (-35 and -10) are highlighted in grey. Known (N16961, [72]) and predicted (O395) half-site operator sequences are highlighted in orange. Known (N16961) and predicted (O395) LexA-binding sites are shown in bold blue and underlined. RstR operators [36] are indicated by green boxes. (B) Alignment of 9 representative *V. cholerae rstR*-*rstA *intergenic regions. The LexA-binding site is shown in a blue box. The alignment shows a clear-cut distinction between El Tor and classical biotypes, but remarkably little divergence within each group, consistent with a recent dissemination of these phages. Sequence alignment was carried out using CLUSTALW [94] and default parameters. Adobe Portable Document File. Click here for file

Additional file 2 **Analysis of *setR *promoter regions**. Alignment of the *setR *promoter region in different *Vibrio cholerae *strains. The SetR-binding sites [95] are highlighted in yellow. The putative promoter elements (-10 and -35) are bolded in blue. The translation start site for *setR *is bolded in red. Sequence alignment was carried out using CLUSTALW [94] and default parameters. Adobe Portable Document File. Click here for file

Additional file 3 **EMSA competition experiments**. The lanes show, respectively, the standard EMSA using *V. parahaemolyticus *LexA and *lexA *promoter, the competition assay adding 200-fold excess of unlabelled lexA promoter, and the competition assay adding 200-fold excess of unlabelled non-specific DNA. JPEG image. Click here for file

Additional file 4 **LexA-binding site searches**. Results for the LexA-binding site searches for the three phylogenetic groups analyzed in this work. The results are organized by organism and gene, providing information on the gene locus identifier (no identifier indicates absence in that organism), the predicted LexA-binding site sequence and its distance to the gene translation start site. Microsoft Excel format. Click here for file

Additional file 5 **Oligonucleotides**. List of all oligonucleotides used in this work. Microsoft Word format. Click here for file

Additional file 6 **Strains and plasmids**. List of all strains and plasmids used in this work. Microsoft Word format. Click here for file
