# Supplementary material for: Analysis of the SOS response of Vibrio and other bacteria with multiple chromosomes
Source: BMC Genomics. 2012 Feb 3;13:58. doi: 10.1186/1471-2164-13-58 (PMC3323433; doi:10.1186/1471-2164-13-58)
Supplement: Additional file 1 — Analysis of rstR promoter regions. (A) Comparison of the rstR-rstA intergenic region of V. cholerae O395 classical O1 serotype and V. cholerae O1 biovar El Tor str. N16961. Predicted (O395) and known (N16961) promoter elements (-35 and -10) are highlighted in grey. Known (N16961, [72]) and predicted (O395) half-site operator sequences are highlighted in orange. Known (N16961) and predicted (O395) LexA-binding sites are shown in bold blue and underlined. RstR operators [36] are indicated by green boxes. (B) Alignment of 9 representative V. cholerae rstR-rstA intergenic regions. The LexA-binding site is shown in a blue box. The alignment shows a clear-cut distinction between El Tor and classical biotypes, but remarkably little divergence within each group, consistent with a recent dissemination of these phages. Sequence alignment was carried out using CLUSTALW [94] and default parameters. Adobe Portable Document File. [file 1471-2164-13-58-S1.PDF]

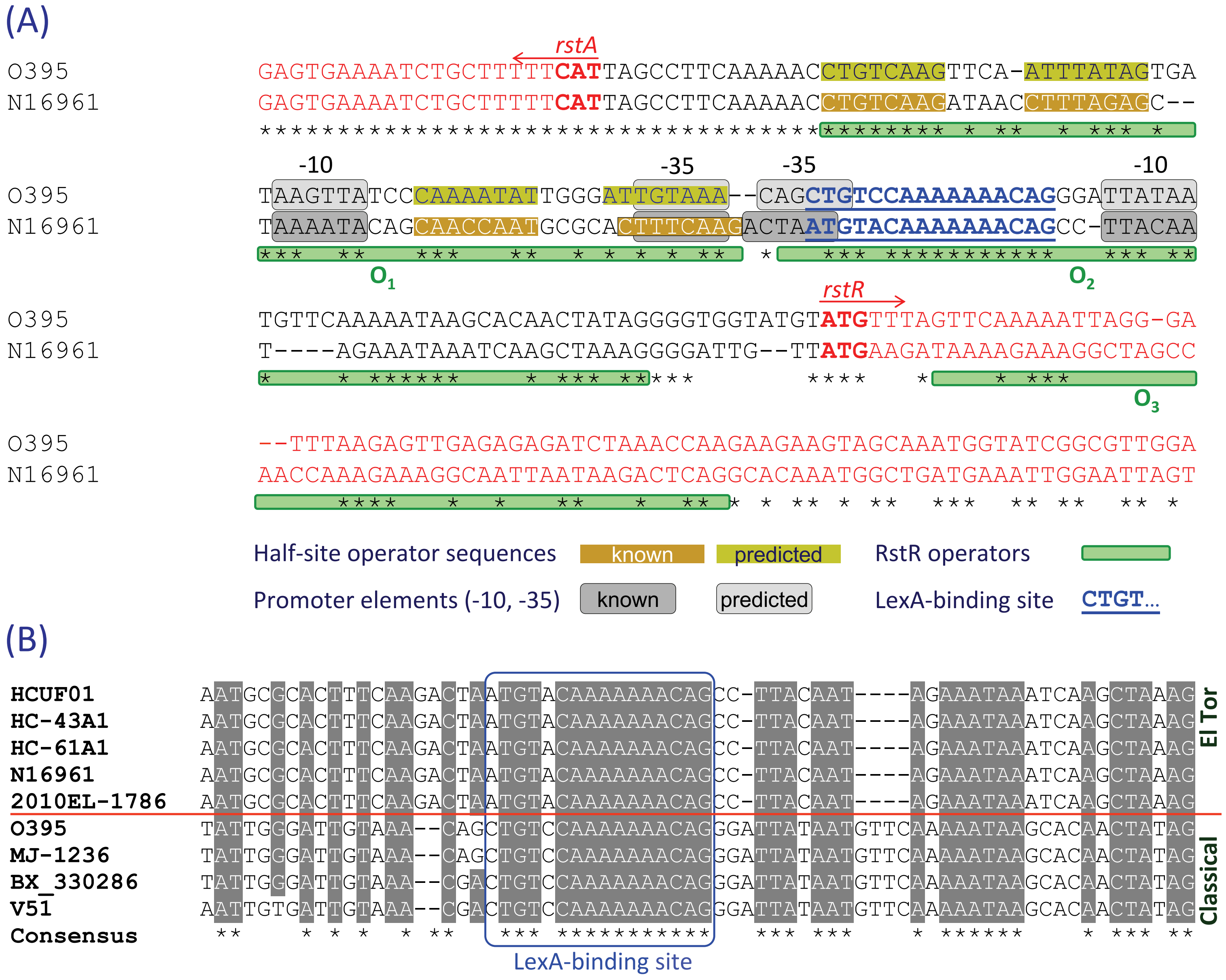
(A)
rstA
O395 GAGTGAAAATCTGCTTTTTCATTAGCCTTCAAAACCTGTCAAGTTCA-ATTTATAGTGA
N16961 GAGTGAAAATCTGCTTTTTCATTAGCCTTCAAAACCTGTCAAGATAACCTTTAGAGC--
-10
-35
-35
-10
O395 TAAGTTATCCCAAAATATTGGGATTGTAAA--CAGCTGTCCAAAAAAACAGGGATTTATAA
N16961 TAAAATACAGCAACCAATGCGCACTTTCAAGACTAATGTACAAAAAAACAGCC-TTACAA
O_1
O_2
rstR
O395 TGTTCAAAAATAAGCACAACTATAGGGGTGGTATGTATGTTTAGTTCAAAAATTAGG-GA
N16961 T----AGAAATAAATCAAGCTAAAGGGGATTG--TTATGAAGATAAAAGAAAGGCTAGCC
O_3
O395 --TTTAAGAGTTGAGAGAGATCTAAACCAAGAAGAAGTAGCAAATGGTATCGGCGTTGGA
N16961 AACCAAAGAAAGGCAATTAATAAGACTCAGGCACAAATGGCTGATGAAATTGGAATTAGT
Half-site operator sequences
known
predicted
RstR operators
Promoter elements (-10, -35)
known
predicted
LexA-binding site
CTGT...
(B)
HCUF01 AATGCGCACTTTCAAGACTAATGTACAAAAAAACAGCC-TTACAAT----AGAAATAAATCAAGCTAAAG
HC-43A1 AATGCGCACTTTCAAGACTAATGTACAAAAAAACAGCC-TTACAAT----AGAAATAAATCAAGCTAAAG
HC-61A1 AATGCGCACTTTCAAGACTAATGTACAAAAAAACAGCC-TTACAAT----AGAAATAAATCAAGCTAAAG
N16961 AATGCGCACTTTCAAGACTAATGTACAAAAAAACAGCC-TTACAAT----AGAAATAAATCAAGCTAAAG
2010EL-1786 AATGCGCACTTTCAAGACTAATGTACAAAAAAACAGCC-TTACAAT----AGAAATAAATCAAGCTAAAG
O395 TATTGGGATTGTAAA--CAGCTGTCCAAAAAAACAGGGATTATAATGTTCAAAAATAAGCACAACTATAG
MJ-1236 TATTGGGATTGTAAA--CAGCTGTCCAAAAAAACAGGGATTATAATGTTCAAAAATAAGCACAACTATAG
BX_330286 TATTGGGATTGTAAA--CGACTGTCCAAAAAAACAGGGATTATAATGTTCAAAAATAAGCACAACTATAG
V51 AATTGTGATTGTAAA--CGACTGTCCAAAAAAACAGGGATTATAATGTTCAAAAATAAGCACAACTATAG
Consensus
El Tor
Classical
LexA-binding site
