# Supplementary material for: Analysis of the SOS response of Vibrio and other bacteria with multiple chromosomes
Source: BMC Genomics. 2012 Feb 3;13:58. doi: 10.1186/1471-2164-13-58 (PMC3323433; doi:10.1186/1471-2164-13-58)
Supplement: Additional file 2 — Analysis of setR promoter regions. Alignment of the setR promoter region in different Vibrio cholerae strains. The SetR-binding sites [95] are highlighted in yellow. The putative promoter elements (-10 and -35) are bolded in blue. The translation start site for setR is bolded in red. Sequence alignment was carried out using CLUSTALW [94] and default parameters. Adobe Portable Document File. [file 1471-2164-13-58-S2.PDF]

```
Ind5     TGGCACGGCGGAGATGTTTTTGTGTTGTGTTGAACACTTCCATACCGTCTCCTGTTACAATAATAACTGT
HFU-02   TGGCACGGCGGAGATGTTTTTGTGTTGTGTTGAACACTTCCATACCGTCTCCTGTTACAATAATAACTGT
HC-48A1  TGGCACGGCGGAGATGTTTTTGTGTTGTGTTGAACACTTCCATACCGTCTCCTGTTACAATAATAACTGT
MO10     TGGCACGGCGGAGATGTTTTTGTGTTGTGTTGAACACTTCCATACCGTCTCCTGTTACAATAATAACTGT
HC-28A1  TGGCACGGCGGAGATGTTTTTGTGTTGTGTTGAACACTTCCATACCGTCTCCTGTTACAATAATAACTGT
B33      TGGCACGGCGGAGATGTTTTTGTGTTGTGTTGAACACTTCCATACCGTCTCCTGTTACAATAATAACTGT
MZO-3    TGGCACGGCGGAGATGTTTTTGTGTCGTGTTGAACACTTCCATACCGTCTCCTGTTACAATAATAACTGT
         ************************* ********************************************

Ind5     TACAAGATTGAATGTTACAGTTTAAACTGTAGATTAGTCAACAGTTAAAATTGTTGAAAGGCTACAGTTT
HFU-02   TACAAGATTGAATGTTACAGTTTAAACTGTAGATTAGTCAACAGTTAAAATTGTTGAAAGGCTACAGTTT
HC-48A1  TACAAGATTGAATGTTACAGTTTAAACTGTAGATTAGTCAACAGTTAAAATTGTTGAAAGGCTACAGTTT
MO10     TACAAGATTGAATGTTACAGTTTAAACTGTAGATTAGTCAACAGTTAAAATTGTTGAAAGGCTACAGTTT
HC-28A1  TACAAGATTGAATGTTACAGTTTAAACTGTAGATTAGTCAACAGTTAAAATTGTTGAAAGGCTACAGTTT
B33      TACAAGATTGAATGTTACAGTTTAAACTGTAGATTAGTCAACAGTTAAAATTGTTGAAAGGCTACAGTTT
MZO-3    TACAAGATTGAATATTACAGTTTAAACTGTAGATGAGTCAACAGTTAAAATTGTTGAAAGACTACAGTTT
         ************* ******************** ************************* *********

Ind5     TATTTGTAGAATACGGGCTTATGAAAACTTTATCCGAACGACTAAACCATGCCTTGCAGCTTACTGGGGT
HFU-02   TATTTGTAGAATACGGGCTTATGAAAACTTTATCCGAACGACTAAACCATGCCTTGCAGCTTACTGGGGT
HC-48A1  TATTTGTAGAATACGGGCTTATGAAAACTTTATCCGAACGACTAAACCATGCCTTGCAGCTTACTGGGGT
MO10     TATTTGTAGAATACGGGCTTATGAAAACTTTATCCGAACGACTAAACCATGCCTTGCAGCTTACTGGGGT
HC-28A1  TATTTGTAGAATACGGGCTTATGAAAACTTTATCCGAACGACTAAACCATGCCTTGCAGCTTACTGGGGT
B33      TATTTGTAGAATACGGGCTTATGAAAACTTTATCCGAACGACTAAACCATGCCTTGCAGCTTACTGGGGT
MZO-3    TATTTGTAGAATACGGGCTTATGAAAACTTTATCCGAACGACTAAACCATGCCTTGCAGCTTACTGGGGT
         **********************************************************************
```
