# Supplementary figures and images for: Analysis of the SOS response of Vibrio and other bacteria with multiple chromosomes
Source: BMC Genomics. 2012 Feb 3;13:58. doi: 10.1186/1471-2164-13-58 (PMC3323433; doi:10.1186/1471-2164-13-58)

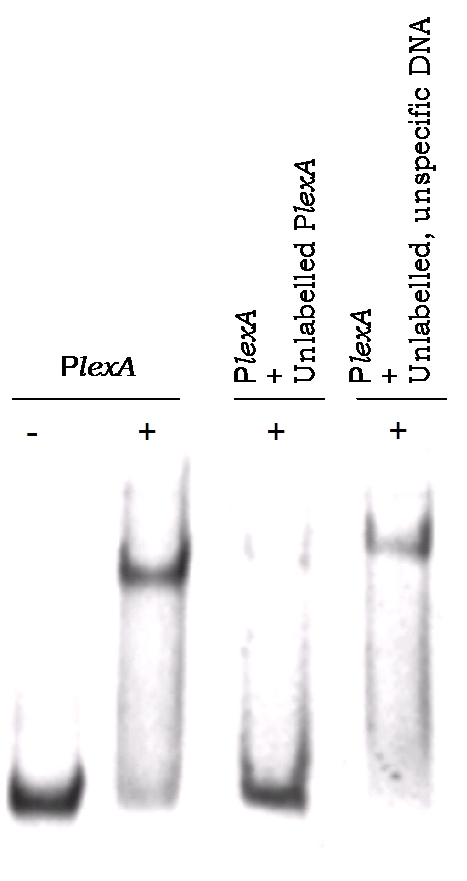

Supplement: Additional file 3 — EMSA competition experiments. The lanes show, respectively, the standard EMSA using V. parahaemolyticus LexA and lexA promoter, the competition assay adding 200-fold excess of unlabelled lexA promoter, and the competition assay adding 200-fold excess of unlabelled non-specific DNA. JPEG image. [file 1471-2164-13-58-S3.JPEG]
