# Supplementary material for: Analysis of the SOS response of Vibrio and other bacteria with multiple chromosomes
Source: BMC Genomics. 2012 Feb 3;13:58. doi: 10.1186/1471-2164-13-58 (PMC3323433; doi:10.1186/1471-2164-13-58)
Supplement: Additional file 5 — Oligonucleotides. List of all oligonucleotides used in this work. Microsoft Word format. [file 1471-2164-13-58-S5.DOC]

| **Oligonucleotides used in this work** | | |
| --- | --- | --- |
| **Name** | **Application** | **Sequence (5’→3’)** |
| NdelexAVpa | Upper primer for cloning the *V.parahaemolyticus* *lexA* gen in pET15b overexpression vector. | CATATGAAGCCGTTAACGCCACGCC^a^ |
| XholexAVpa | Lower primer for cloning the *V.parahaemolyticus* *lexA* gen in pET15b overexpression vector | CTCGAGTTACATCCAATCGGTATTG^a^ |
| recAVpaF | Synthetic oligo to obtain the RecA EMSA probe. | TCATACAGGTATAGACACTGTATGAATCAACAGTATAATGACTTTCATTGCTGAGCAGAAA |
| recAVpaR | Synthetic oligo to obtain the RecA EMSA probe. | CAATGAAAGTCATTATACTGTTGATTCATACAGTGTCTATACCTGTATGAAAAAAATTTGA |
| lexAVpaF | Synthetic oligo to obtain the LexA EMSA probe. | GATATACTCACAGTTAACTGTATAAAAAGACAGGTGAGACATGAAGCCGTTAACGCCACGA |
| lexAVpaR | Synthetic oligo to obtain the LexA EMSA probe. | CGTGGCGTTAACGGCTTCATGTCTCACCTGTCTTTTTATACAGTTAACTGTGAGTATATCA |
| recGVpaF | Synthetic oligo to obtain the RecG EMSA probe. | TTTCTACGCCACTTCTTATAGTTTTTCCTGTACAAAAACACAGCTCAATGGTTAACATACTGCTATGTTAA |
| recGpaR | Synthetic oligo to obtain the RecG EMSA probe. | TAACATAGCAGTATGTTAACCATTGAGCTGTGTTTTTGTACAGGAAAAACTATAAGAAGTGGCGTAGAAAA |
| mutHVpaF | Synthetic oligo to obtain the MutH EMSA probe. | GCCTAAAAACGTTTCAAAACCCCTGTTTATTCATCCAGCCCATCAGTAGATCCACTTATAA |
| mutHVpaR | Synthetic oligo to obtain the MutH EMSA probe. | TATAAGTGGATCTACTGATGGGCTGGATGAATAAACAGGGGTTTTGAAACGTTTTTAGGCA |
| imuAVpaF | Synthetic oligo to obtain the ImuA EMSA probe. | GTGTTTTCATCATAGAAATATACTGTATTTATATACAGGTATTTTATTTATGCAAGACATA |
| imuAVpaR | Synthetic oligo to obtain the ImuA EMSA probe. | ATGTCTTGCATAAATAAAATACCTGTATATAAATACAGTATATTTCTATGATGAAAACACA |
| topBVpaF | Synthetic oligo to obtain the TopB EMSA probe. | ATACATACCTAGATAACGCTTACTGTTCATTTATACAGTTTTTTCTTGATTTCTTAGGGCA |
| topBVpaR | Synthetic oligo to obtain the TopB EMSA probe. | GCCCTAAGAAATCAAGAAAAAACTGTATAAATGAACAGTAAGCGTTATCTAGGTATGTATA |
| unfAVpaF | Synthetic oligo to obtain the UnfA EMSA probe. | ATCAGATACCCAAAACAAACTACTGTATACACATACAGCATGTATAAAGGAACAGTAAGAA |
| unfAVpaR | Synthetic oligo to obtain the UnfA EMSA probe. | TCTTACTGTTCCTTTATACATGCTGTATGTGTATACAGTAGTTTGTTTTGGGTATCTGATA |
| unfBVpaF | Synthetic oligo to obtain the UnfB EMSA probe. | TAGGAGGAAATTATAAACAATACTGTTTTTATATACAGTATCTAGTTTGGAGGTGAAGTAA |
| unfBVpaR | Synthetic oligo to obtain the UnfB EMSA probe. | TACTTCACCTCCAAACTAGATACTGTATATAAAAACAGTATTGTTTATAATTTCCTCCTAA |
| M13F/pUC | Universal upper primer of pGEMT vector to obtain the EMSA probe labeled with Digoxigenin (DIG). | DIG/gttttcccagtcacgac |
| M13R/pUC | Universal lower primer of pGEMT vector to obtain the EMSA probe labeled with Digoxigenin (DIG). | caggaaacagctatgac |
