# Supplementary material for: Analysis of the SOS response of Vibrio and other bacteria with multiple chromosomes
Source: BMC Genomics. 2012 Feb 3;13:58. doi: 10.1186/1471-2164-13-58 (PMC3323433; doi:10.1186/1471-2164-13-58)
Supplement: Additional file 6 — Strains and plasmids. List of all strains and plasmids used in this work. Microsoft Word format. [file 1471-2164-13-58-S6.DOC]

| **Strains and plasmids used in this work** | | |
| --- | --- | --- |
| **Name** | **Relevant characteristics genotype** | **Reference** |
|  |  |  |
| **Strains** | | |
| ATCC17802 | *V. parahaemolyticus* ATCC17802 soca salvatge | From ATCC collection |
| K12 | *E. coli* K12 soca salvatge | From DSMZ collection |
|  |  |  |
| **Plasmids** | | |
| pET15b | Overexpression vector that carries an N-terminal His·Tag to allow purification of the *V.parahaemolyticus* *lexA* gen. | Novagen (Cat. No. 69661-3) |
| pGEMT | Cloning vector pGEM-T System I used to obtain the EMSA probes labeled with Digoxigenin. | Promega (Cat. No. A3600) |
